# Supplementary material for: End Sequence Analysis Toolkit (ESAT) expands the extractable information from single-cell RNA-seq data
Source: Genome Res. 2016 Oct;26(10):1397–410. doi: 10.1101/gr.207902.116 (PMC5052061; doi:10.1101/gr.207902.116)
Supplement: Supplemental Material [file supp_gr.207902.116_Supp_Materials.tar.gz › ESAT-esat_v0.1/doc/html/functions_vars_m.html]

ESAT: Class Members - Variables


ESAT


- Main Page
- Related Pages
- Packages
- Classes
- Files

- Class List
- Class Index
- Class Hierarchy
- Class Members

- All
- Functions
- Variables
- Enumerator

- a
- b
- c
- d
- e
- f
- g
- h
- i
- k
- l
- m
- n
- o
- p
- q
- r
- s
- t
- u
- v
- w


All Classes Namespaces Files Functions Variables Enumerator Pages

### - m -

- MAIN\_ARRAY\_LEFT
  : broad.pda.seq.rap.CountProbes
- MAIN\_ARRAY\_RIGHT
  : broad.pda.seq.rap.CountProbes
- MASK\_FILE
  : broad.pda.seq.rap.GenomeCommandLineProgram
- match
  : umms.core.annotation.PSLRecord
- MATCH\_OPPOSITE\_STRAND
  : broad.pda.seq.rap.DistanceToBED
- mateCigarFlag
  : umms.core.writers.PairedEndWriter
- mateLineFlag
  : umms.core.writers.PairedEndWriter
- mateSequenceFlag
  : umms.core.writers.PairedEndWriter
- MAX\_DISTANCE
  : umms.core.annotation.AbstractAnnotation
- MAX\_FRAGMENT\_LENGTH
  : broad.pda.seq.rap.GenomeCommandLineProgram
- MAX\_INSERT
  : broad.pda.samtools.PairedEndTDF
  , broad.pda.samtools.SAMPairedEndFileReader
- maxDist
  : broad.pda.differentialExpression.FWERDistribution
- maxFragmentLengthData
  : broad.pda.seq.clip.SampleData
  , broad.pda.seq.protection.SampleData
- maxFragmentLengthGeneScores
  : broad.pda.seq.clip.SampleData
- METRICS
  : broad.pda.samtools.FilterPairedReadsByMAQ
- MIDDLE\_LENGTH
  : broad.pda.seq.rap.PlotAggregateRegions
- MIN\_MAPPING\_QUALITY
  : broad.pda.seq.rap.GenomeCommandLineProgram
- MIN\_WINDOW\_COUNT
  : broad.pda.seq.protection.TwoSampleScanSkellamPeakCaller
- minDist
  : broad.pda.differentialExpression.FWERDistribution
- MINIMUM\_MAPPING\_QUALITY
  : broad.pda.samtools.FilterPairedReadsByMAQ
- MISMATCH
  : jaligner.Markups
- MmeI
  : broad.core.primer3.RestrictionEnzyme
- MRNA\_ARRAY\_LEFT
  : broad.pda.seq.rap.CountProbes
- MRNA\_ARRAY\_RIGHT
  : broad.pda.seq.rap.CountProbes


---

Generated on Fri Aug 15 2014 15:11:17 for ESAT by  

 1.8.7
